# New library construction method for single-cell genomes

**DOI:** 10.1371/journal.pone.0181163

**Published:** 2017-07-19

**Authors:** Larry Xi, Alexander Belyaev, Sandra Spurgeon, Xiaohui Wang, Haibiao Gong, Robert Aboukhalil, Richard Fekete

**Affiliations:** Fluidigm Corporation, South San Francisco, California, United States of America; University of Helsinki, FINLAND

## Abstract

A central challenge in sequencing single-cell genomes is the accurate determination of point mutations, phasing of these mutations, and identifying copy number variations with few assumptions. Ideally, this is accomplished under as low sequencing coverage as possible. Here we report our attempt to meet these goals with a novel library construction and library amplification methodology. In our approach, single-cell genomic DNA is first fragmented with saturated transposition to make a primary library that uniformly covers the whole genome by short fragments. The library is then amplified by a carefully optimized PCR protocol in a uniform and synchronized fashion for next-generation sequencing. Each step of the protocol can be quantitatively characterized. Our shallow sequencing data show that the library is tightly distributed and is useful for the determination of copy number variations.

## Introduction

The genetic variations that can occur in single cells, such as single-nucleotide variations (SNVs) and copy number variations (CNVs) are the driving forces in many biological processes, including evolution and cancer [1]. Most of the current studies on genetic variations rely on bulk DNA sequencing, which only provides a coarse view into the average state of a population of cells. Although bulk sequencing provides an adequate picture for studies at the germline level or for homogeneous systems, it works poorly for systems such as solid tumors, which are complex mixtures of cells that can include noncancerous fibroblasts, endothelial cells, lymphocytes, and macrophages. The noncancerous cells can contribute more than 50% of the total DNA extracted, potentially masking important aberrations from the cancer cells [2]. In addition, the heterogeneity of cancerous cells within tumors and the myriad of genome instability processes that shape tumor evolution over space and time cannot be resolved by bulk sequencing [3, 4]. In contrast, single-cell approaches using next-generation sequencing (NGS) have yielded important insights into the key genomic features of various subpopulations and the evolutions of various cancer clones [5].

The quantity of genetic material that can be isolated from a single cell is inadequate for NGS for many applications, which means that the whole genome needs to be amplified prior to sequencing. This amplification process must generate sufficient quantities of DNA with minimal dropout to ensure a good representation of the whole genome. The amplification also needs to be unbiased and uniform to ensure maximal coverage with minimal sequencing effort. In recent years, several labs have reported various single-cell whole genome amplification and library preparation methods, including multiple displacement amplification (MDA) [6–10], degenerate oligonucleotide-primed PCR (DOP-PCR) [11, 12], and multiple annealing and looping-based amplification cycles (MALBAC) [13]. MDA uses degenerate nucleotides to initiate amplification by phi29 DNA polymerase in an isothermal process to randomly generate copious genomic DNA [14]. DOP-PCR utilizes a pool of tagged semi-random primers to amplify the whole genome in two stages. The first few cycles of PCR are done at low temperature to enhance random priming, and then amplification is continued at higher annealing temperature unsuitable for random priming, but suitable for the specific primer priming the tag sequence [11, 12]. MALBAC uses a pool of primers, each having 8 variable nucleotides connected to a common 27-nucleotide tail at its 5ʹ end. The first several amplification cycles proceed in a quasi-linear manner, and then at higher annealing temperature the amplification enters exponential phase using the common 27-nucleotide tail of the primer [13].

Although proven useful, these methods have their respective limitations; they tend to preferentially amplify some areas of the genome over others in different ways. DOP-PCR became the choice for the detection of CNVs, while MDA is preferred method for SNV [15]. We feel that there is need for a new single-cell library preparation method that suits both purposes. Toward that ultimate goal, we present a new way to construct single-cell genome libraries using the Transposon Barcoded (TnBC) library. In this paper we first describe how this library is constructed and explain how the unique fragment identifier (UFI) is generated. We then describe and discuss the factors that affect library amplification and explain the characterization of the libraries using qPCR, UFI, and shallow sequencing. We elaborate methods in quantitatively assessing the library. Finally, we demonstrate how shallow sequencing of these libraries can be used to trace copy number changes during cell culture.

While we were preparing this manuscript, two new methods, DLP [16] and LIANTI [17], were reported. Like TnBC, both DLP and LIANTI transpose single cell genomic DNA directly. The DLP libraries are sequenced without any amplification. Due to the way that Nextera is constructed, only 50% of the genome can be sequenced. As a result, DLP is suitable only for CNV detection in single cells [16]. Like TnBC, LIANTI amplifies single cell library before sequencing, except it employs linear amplification. Unlike TnBC, LIANTI does not take measures to ensure the majority of the inserts of the library are short enough so that the inserts can be completely covered by Illumina NGS; no UFI information was extracted [17].

## Materials and methods

### Titration of transposase in tube reactions

In-tube transposition reactions were carried out in 16.7 μL 1X MuSeek Fragmentation Reaction Buffer (MuSeek^™^ Library Preparation Kit, Illumina^™^ compatible, cat. no. K1361 by Thermo Scientific^™^, Waltham, MA, USA) containing 4.44 ng of purified human DNA (cat. no. D1234152, BioChain^®^, Newark, CA, USA) and 0.023 μL, 0.047 μL, 0.093 μL, 0.19 μL, 0.38 μL, 0.75 μL, 1.5 μL, and 3 μL of Mu transposase (from the tube labeled as MuSeek Enzyme Mix, IL in cat. no. K1361 by Thermo Scientific), respectively. Transpositions were performed at 30°C for 30 minutes, and then stopped by adding 6.7 μL solution containing 34.4 mM EDTA and 0.09 unit/μL Proteinase K (New England Biolabs, Ipswich, MA, USA). The mixes were incubated at 55°C for 30 minutes to allow the degradation of Mu transposase by Proteinase K and another 20 minutes at 70°C to denature Proteinase K. An aliquot of 3.1 μL of each of above libraries was amplified in 30 μL PCR mix containing 1X Phusion Hot Start II High-Fidelity PCR Master Mix (cat. no. F-565L, Thermo Fisher), 1.8 μg of chymostatin (Sigma-Aldrich^®^ Co., St Louis, Mo, USA), 100 μM of MuEnd Primer (CGTTTTTCGTGCGTCAGTTCA, Integrated DNA Technologies, Coralville, Iowa, USA) and 1X EvaGreen^™^ (Biotium, Fremont, CA, USA). The profile of thermal cycling was as follows: 30 minutes at 75°C, 2 minutes at 98°C, 12 minutes at 70°C, followed by 11 cycles between 30 seconds at 98°C and 6 minutes at 70°C. The amplified product was purified with AMPure beads (Beckman Coulter, Brea, CA, USA) by following manufacturer’s instruction and analyzed on Agilent 2100 Bioanalyzer with Agilent High Sensitivity DNA Analysis Kit (Agilent, Santa Clara, CA, USA).

### Library barcoding and NGS

A purified MuEnd-amplified library, either from a single cell or purified DNA, was barcoded using PCR in 1X Phusion Hot Start II High-Fidelity PCR Master Mix containing 1X EvaGreen and 500 nM each of barcoding primers following the instruction of MuSeek Library Preparation Kit, Illumina compatible. Barcoded libraries were purified with AMPure beads and quantified based on the electropherogram obtained on the Agilent 2100 Bioanalyzer with Agilent High Sensitivity DNA Analysis Kit before they were sequenced on MiSeq^™^ using custom primers provided by the MuSeek Library Preparation Kit.

### Alignment and processing of sequence data

**Fig 1:**
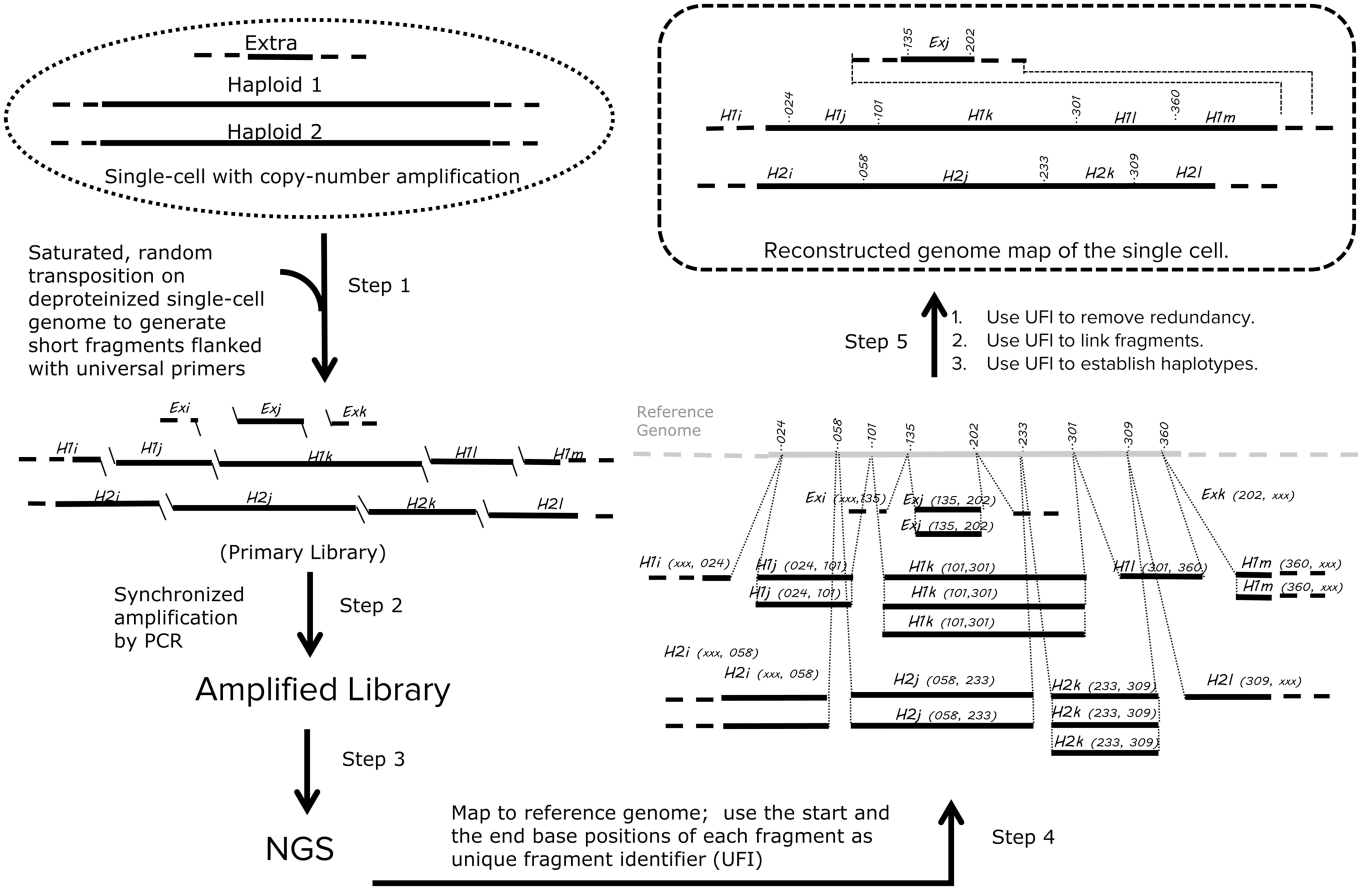
TnBC workflow. After lysis of a single cell (which consists of two haploids and one extra copy in the figure for illustration) and removal of histone proteins, the exposed genome is transposed under a saturating concentration of loaded transposase, generating a library that consists of short fragments flanked with universal primers (Step 1). The fragments derived from Haploid 1 are marked as H1i, H1j, H1k, H1l, and H1m. Similarly, the fragments derived from Haploid 2 and the extra copy are marked as H2i, H2j, H2k, H2l, Exi, Exj, and Exk respectively. The library is then amplified synchronously using the universal primer (Step 2) and the amplified library is converted to an NGS-compatible library and sequenced by NGS (Step 3). In Step 4, each sequences read is mapped to the reference genome, and the start and the end bases of each fragment are registered as UFI for that fragment. For example, the UFI of fragment H1k is a pair of positions on the reference (…101, …301). For simplicity, only the last three digits are shown. In Step 5, redundant fragments, such as three H1k (…101, …301) are identified and consolidated into one unique fragment. UFIs of H1j (…024, …101), H1k (…101, …301), and H1l (…301, …360) are used to link the fragments. Although H1k, H2j, and Exi are mapped to the same region of the reference genome, their UFIs show that they are exclusive from each other, so we conclude that they originated from three different physical copies. The exact location of Exi may be eventually determined when the coverage is high enough. When the coverage is less than 1X genome coverage, the UFI can be used to eliminate amplification duplication for CNV calls.

Paired-end sequencing reads were aligned to GRC37/hg19 reference using bowtie2 (2.2.4) with the command $bowtie2 -L 30 -X 3000 -x $genome_index -1 $read1–2 $read2 -S $sam_file [18, 19]. Reads with mapping score <20, reads of size over 500 bp, and reads that have multiple hits to the reference genome were removed. A UFI was assigned to each read using its exact start and end positions in the coordinate of the reference genome (Fig 1). Two or more reads with identical UFI were consolidated to a single read. Genome coverage was calculated based on read counts or unique reads by bedtools (2.17.0) [20, 21]. CNV calls were made using Ginkgo [22].

### Loading Mu18

Mu transposase loaded with engineered transposon ends, Mu18, was prepared by following the procedure disclosed by Ukanis et al. (Ukanis et al. US2016/0177359). Specifically, in a loading buffer that contained 120 mM Tris, pH 8.0, 100 mM NaCl, 0.05% Triton X-100, 1 mM EDTA, 10% glycerol, and 10% DMSO, unloaded Mu transposase (custom order from Thermo Fisher) at final concentration of 1.65 mg/mL was mixed with triplex formed by MuT (TGCTGAACTGACGCACGAAAAACGCGAAAGCGTTTCACGATAAATGCGAAAAC), Mu18b (TTTTCGTGCGTCAGTTCA), and Mu18e (GTTTTCGCATTTATCGTGAAACGCTTTCGCGT) at final concentration of 4.7 μM. Loading was allowed to proceed at 4°C overnight. Although different nick positions were listed in the patent disclosure, Mu18 appeared to be slightly more active.

### The impacts of lengths of the terminal repeat and the inserts on PCR efficiency

Four synthetic single-stranded DNA templates were synthesized (Integrated DNA Technologies), representing amplification targets. All templates follow the primary structure of terminal repeat—insert—terminal repeat and they could form stem-loop structures at proper conditions. Each template was denoted by the lengths of stem and loop in the form of stem/loop. Four templates are 50/300, 50/50, 21/300, and 21/50 respectively. PCR amplifications of 8 pM single-stranded templates were carried out on ABI Prism^®^ 7900HT Real-Time PCR System in 1X Phusion Hot Start II High-Fidelity PCR Master Mix, 2% ROX reference dye (Invitrogen^™^, P/N 54881), 1X EvaGreen, and Primer MuEnd at 500 nM or at 10,000 nM by an initial 30-second incubation at 98°C followed by 35 cycles between 10 seconds at 98°C, 40 seconds at 59°C, and 30 seconds at 72°C.

### Cells and single-cell isolation and TnBC library construction in tubes

BJ and K562 cell lines were purchased from ATCC^®^ (Manassas, VA, USA). The human lymphocyte GM01202 cell line was from Coriell Institute (Camden, NJ, USA). Reagents were purchased from Thermo Fisher Scientific and their subsidiaries (San Diego, CA) unless noted otherwise. For cell sorting into a 96-well plate, cells were harvested and washed once in DMEM and resuspended at 1 million cells/mL. Cell sorting was performed using a 100 μm sorting chip on Sony SH800 cell sorter (Sony Biotechnologies). The machine was carefully optimized to achieve maximal purity and viability for cells. Cells were sorted for cell size and cell complexity using forward scatting light and back scatting light. Doublets were excluded using FSC width and single cells were sorted using single-cell mode in 96 plates containing 5μL of Mu transposition buffer (40 mM Tris, pH 8.0, 0.33 mM EDTA, 100 mM NaCl, 10 mM MgCl_2_, 0.05% TritonX-100, 10% glycerol, and 3.3% DMSO) containing 0.033 unit of Proteinase K. The plates were immediately centrifuged at 3220 x *g* for 5 minutes and then incubated at 55°C for 30 minutes to lyse the cell and remove histone proteins. Proteinase K was then denatured at 65°C for 20 minutes, one μL of Mu Transposition buffer containing 0.12 mg/mL chymostatin was added to each reaction to inhibit any remaining activity of Proteinase K. Exposed genomic DNA was tagmented by adding 0.6 μL of Mu18 or native Mu transposase from the kit and keeping the reaction at 30°C for 30 minutes or overnight. The resulting TnBC library was mixed with 3.4 μL of a mixture comprised of 0.089 unit/μL of Proteinase K and 34.4 mM EDTA and kept at 55°C for 30 min, followed by heating at 75°C for 20 min. The whole library was amplified by MuEnd primer in a mix that contained 1X Phusion Hot Start II High-Fidelity PCR Master Mix, 2.8 μg of chymostatin, 100 μM of MuEnd Primer, and 1X EvaGreen in a final volume of 50 μL. The reaction was first incubated for 30 minutes at 75°C to extend the nick, followed by 2 minutes at 98°C, 12 minutes at 70°C, and 7 cycles of two-step PCR between 30 seconds at 98°C and 6 minutes at 70°C.

### Single-cell capture and TnBC construction and amplification in Fluidigm^®^ C1^™^ integrated fluidic circuit (IFC)

Freely cultured cells were singulated and washed 5 times with C1 DNA Seq Cell Wash Buffer. Cells were counted and loaded onto C1 Single-Cell Open App IFC (Fluidigm cat. no. 100–8133) according to the manufacturer’s instructions. LIVE/DEAD^®^ viability stain (Invitrogen) was included to enable imaging of the capture sites after cell loading and capture. All capture sites were imaged using a Leica^®^ microscope where phase contrast and fluorescent images with GFP and Y3 filters were acquired to determine the number of cells captured, as well as the viability of each of the captured cells. The cells then were lysed and genomic DNA was fragmented with Mu transposase at the same enzyme to DNA ratio as in the tube tagmentation reaction and amplified using the MuEnd primer by keeping the same concentrations of primer and master mix as in the tubes.

## Results and discussion

### Library preparation

#### The TnBC workflow

The TnBC workflow is illustrated in Fig 1. After cell lysis and histone removal, the single-cell genome is transposed with a saturating concentration of loaded transposase, generating a primary library that consists of short fragments flanked with transposon ends. This process is referred as tagmentation [23]. The library is then amplified synchronously using a universal primer that primes at the transposon ends. Next, the amplified library is converted to an NGS-compatible library and sequenced by NGS. The sequence reads are mapped to the reference genome, and the very start and the very end base positions of each fragment are jointly registered as the UFI of that fragment. Redundant fragments are identified by the UFI and consolidated. The unique fragments are used for downstream bioinformatics analysis.

#### Saturated transposition

Unique to the TnBC protocol is the tagmentation of the single-cell genome to make a primary library in the first step before an amplification step (Fig 1). Tagmentation attaches a universal priming sequence, which allows every fragment from the single cell genome, regardless of its GC content, an equal chance to be primed throughout the amplification process [23]. In contrast, MDA utilizes random priming, thereby allowing bias to accumulate throughout the process [14]. On the other hand, DOP-PCR and MALBAC limit the use of random priming to the first stage, consisting of only a few initial amplification cycles, and shift to cycling with universal primers in the second stage [12, 13].

**Fig 2:**
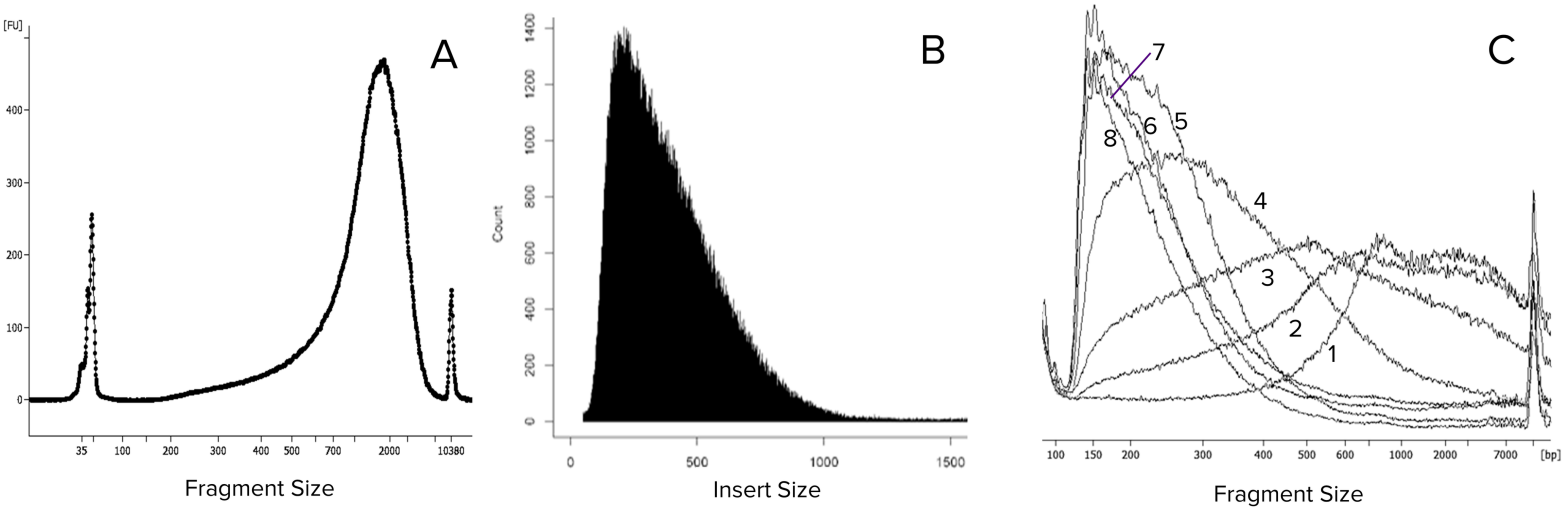
Size preference of NGS and titration of transposase. Profiles of an NGS library are compared: Panel A shows what was loaded onto MiSeq (analyzed by Bioanalyzer) and Panel B shows what was actually sequenced. Short fragments are more efficient in forming clusters. Panel C shows profiles of the libraries generated with various ratios of transposon to DNA. Traces 1–8 represent tagmentation reactions of 4.44 ng of human DNA by 0.023 μL, 0.047 μL, 0.093 μL, 0.19 μL, 0.38 μL, 0.75 μL, 1.5 μL, and 3 μL of Mu transposon respectively (details in Materials and methods). Reactions represented by Traces 6, 7, and 8 were under saturating conditions.

Conventional transposition (instruction of MuSeek Library Preparation Kit) generates libraries comprised of fragments ranging from 150 to 2000 bp. We fear that a single-cell library with this profile may not be evenly covered, because fragments <500 bases are reported to have a better chance to be sequenced using paired-end Illumina technology than larger fragments because the shorter ones are more efficient in generating clusters [24]. This finding was confirmed by an experiment shown in Fig 2. Panel A shows a size histogram for a NGS library with a median fragment size of about 1K. This library generated a batch of sequenced fragments with a median size of 400 bp (Panel B). Although larger fragments in a library consisting of small and large fragments could be recovered using deeper coverage, we reasoned that it is much more economic to use libraries exclusively comprised of smaller-size fragments. We employed saturated transposition to ensure that the majority of the genome was tagmented into fragments no larger than 500 bp.

The small fragment size was achieved by adjusting the ratio of transposase to the target DNA as shown in Fig 2C. When the amount of target DNA was held constant as the concentration of transposase was increased, the number of large fragments decreased. When a saturating concentration of transposase was reached, a further increase in the concentration of transposase no longer led to significant change in fragment sizes. We refer these reactions to as saturated transposition. Under our experimental conditions of saturated transposition most of the fragments were smaller than 500 bases, as represented by Traces 6, 7, and 8 in Fig 2C. This is the condition under which two transposase molecules bind to the DNA with variable distance between them but the space between them does not allow a third transposase to squeeze in. Saturated transposition requires high transposase concentrations, which were conveniently achieved using commercially available Mu transposase.

The smallest fragments remained the same size under all conditions, even under saturated concentrations of transposase. The smallest fragment is possibly generated by two loaded transposase molecules binding side by side. After transposition, Mu stays on the product DNA; therefore, tagmented fragments will not be further transposed by fresh transposases. The larger size of Mu transposase relatively to Tn5 transposase results in larger minimal size (50 bases vs. 35 bases by Tn5), which in turn benefits downstream genome mapping. The largest fragment sizes of reactions represented by Traces 7 and 8 were larger than we expected. We speculate that this might be caused by either a small portion of transposase that were active in binding but inactive in tagmentation or due to incomplete removal of histones.

No biases in DNA sequence coverage were reported in NGS library construction by tagmentation [23, 24]. We took notes that preferences for the recognition sequences of transposases were observed under conditions where the concentration of transposase were far less relative to target DNA [25]. Because saturated transposition employs higher transposase concentrations than those used in conventional NGS library constructions, we reasoned that bias in coverage should be less of a concern. Even if there were some preferences on the part of the transposase, the “left over” region in the genome after the initial round of tagmentation in the genome will eventually be transposed.

Conceivably, saturated Mu transposition should generate an unamplified library of perfect 1X coverage of the whole genome, with insert sizes between 50 to 300 bases. If the mean of the insert size is 150 bases, a genome of a single cell of 6,000,000,000 bases should be expected to generate a 1X unamplified library comprised of 20,000,000 individual molecules. This number is the base for quantification using qPCR (details in Evaluation of TnBC libraries).

#### Uniform amplification and modified transposon

The primary library needs to be faithfully amplified before it can be fully characterized by NGS. Although the primary library can be sequenced without amplification [16], the full power of single-cell sequencing was not realized [26, 27]. It is desirable that amplification is accomplished in an unbiased and even manner, with relative copy number unchanged regardless of the size and GC content of the insert. Uniformly small inserts make unbiased amplification easier (Real-time PCR handbook, http://find.thermofisher.com/Global/FileLib/qPCR/real-time-pcr-handbook.pdf). Considering that the two-primer approach used by Illumina for conventional library preparation only amplifies half of the library, we chose single-primer amplification to recover the whole library, as recommended by the MuSeek kit.

However, the relative amplification efficiency of single-primer PCR is sensitive to the lengths of insert and transposon end, because the identical ends in an amplicon tend to form hairpins in PCR [28]. To minimize the difference in amplification efficiency, we shortened the effective length of terminal repeats in the transposon end sequence from 50 bp to 18 bp (Materials and methods). Furthermore we optimized PCR conditions for the full range of inserts yielded by saturated tagmentation.

To demonstrate the equal amplification efficiency by the new system, we employed a set of four model templates that differ in the length of the terminal repeats and the length of the insert. These templates are identified as 50/50, 50/300, 21/50, and 21/300 (Materials and methods).

**Fig 3:**
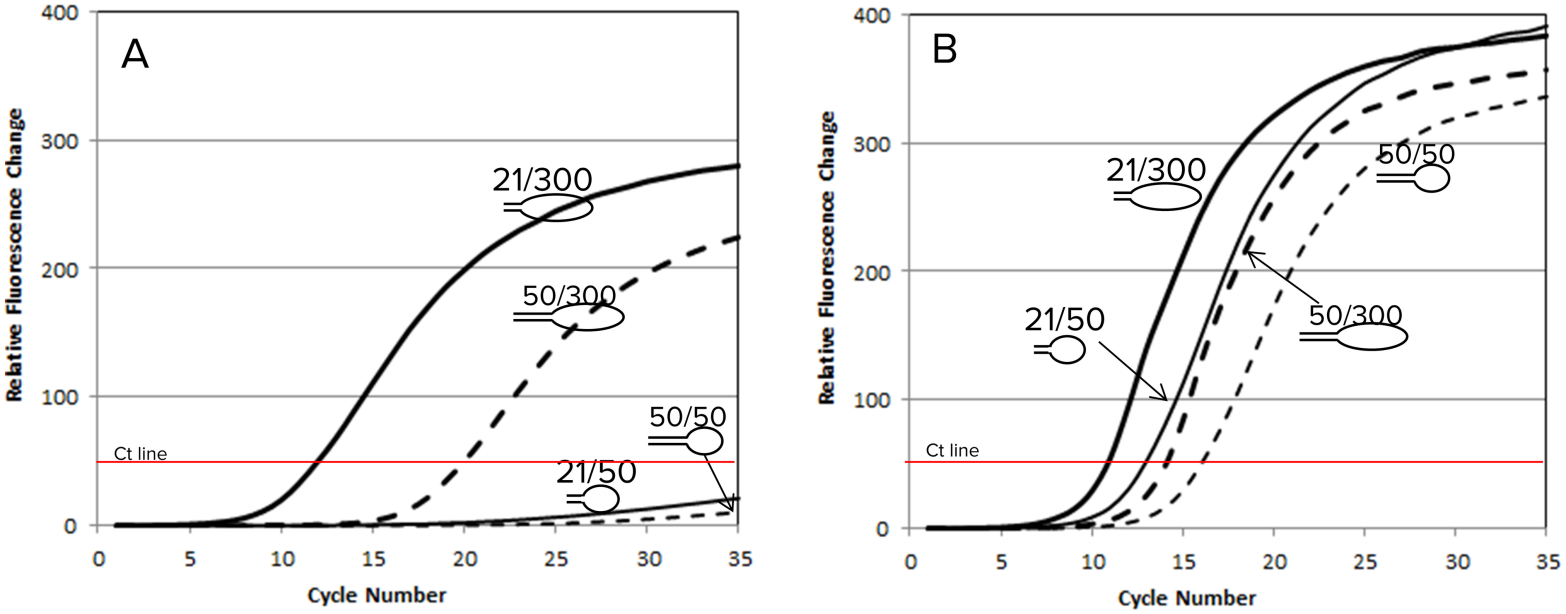
Stem length, insert length, and primer concentration affect PCR amplification. Panel A shows that the efficiency of PCR amplification was affected by either longer stem or short insert under low primer conditions. Once the primer concentration was increased to 10,000 nM, amplification of short insert or long stem improved (Panel B). Still, the templates with shorter stem were more efficient than those of long stems. The short and long insert flanked by short terminal repeat were equally efficient when considering the difference in fluorescence between two double-stranded templates.

Fig 3A shows the amplification curves of the four templates using conditions suggested by the MuSeek kit. With equal concentration of input template, the longer amplicon was favored over the shorter amplicon regardless of the length of the inverted terminal repeat. However, the amplicon with shorter inverted terminal repeats, which mimics templates generated by engineered Mu, was favored over the one with longer terminal repeats, the same length generated by wildtype Mu, for both amplicon lengths. Under the optimized amplification conditions the shorter inserts were amplified practically as efficiently as their longer counterparts (Fig 3B). Furthermore, by using Mu18, we can improve the efficiency by nearly three cycles compared to the wild type transposon. The significance of three-cycle improvement cannot be overemphasized for TnBC libraries. A three-cycle delay means that at maximum, only one eighth of the library generated by the wild type Mu is duplicated in the first cycle. After three cycles, some regions of the genome are present at 8 copies while other regions remain as 1 copy, generating a very prominent bias against the later amplified copies. MuEnd Primer was slightly more efficient than Mu18b as amplification primer; the concentration of MuEnd Primer could be as high as 100 μM without any adverse effects.

### Evaluation of TnBC libraries

#### Evaluation the library by Ct, product profile and UFI

To better evaluate the TnBC library, EvaGreen was added to the amplification mix to monitor the amplification process. A delayed amplification would reflect a low amount of amplifiable inserts in the primary library, indicating either incomplete removal of histone proteins or incomplete removal of transposase.

Whether the transposition reached to saturation can be verified by size profiling the amplified library using electrophoresis, as shown in Fig 2C. Traces 6, 7, and 8 were accepted as saturated transpositions.

Insufficient mixing in transposition may yield long inserts along with short optimal inserts. Amplification of this mixture may also be delayed due to the low efficiency of long inserts. Unfortunately, the electropherogram does not detect failures in amplification of the large fragments. We routinely use 20,000,000 molecules of synthetic oligo of 150 bp long flanked with identical 18-base inverted repeats in a separate reaction tube as an input control.

Once sequenced, the TnBC library can be evaluated by the duplication rate, which is defined as ratio of total reads divided by the total UFI. Of the same library, as the reads number increase, the duplication rate increase. Current TnBC libraries of 1,000,000 reads usually have duplication rate of 1.1. In our experience, high duplication rate are associated with either late Ct, nonsaturated transposition or non-optimal PCR protocols.

#### Evaluation of the uniformity by Lorenz curve

Using saturated transposition and uniform amplification, TnBC single-cell libraries of BJ, GM01202, and K562 were constructed and then sequenced at 0.05X coverage on the MiSeq system. The uniformity across the genome of these libraries was first evaluated against bulk genome libraries constructed from commercial DNA and with the sequencing data (SRR1006146 for DOP-PCR, SRR975229 for MALBAC, and SRR504711 for MDA) downloaded from NCBI. Reads from all samples were mapped to the human genome and down-sampled to 1,000,000 reads. Aligned reads were binned into 50, 100, 250, 500, 1000, 2500, 5000, and 10,000 kb variable-length intervals across the genome with averages of 20, 40, 100, 200, 400, 1000, 2000, and 4000 reads per bin respectively. Lorenz curves of all samples/methods were plotted using various bin sizes (S1 Fig). It is apparent that the larger the bin size is, the closer the curves are to perfect uniformity.

**Fig 4:**
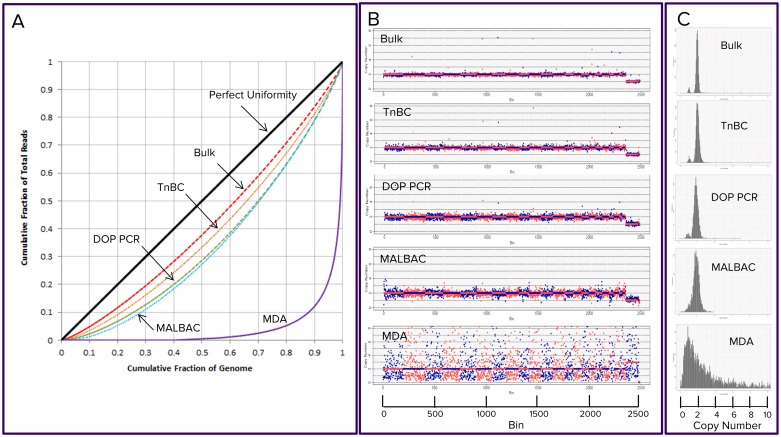
Comparison of different library preparation methods using Lorenz curves, copy number calls, and frequency of bin counts. Lorenz curves of bulk genomic DNA and of single cells by TnBC, DOP-PCR, MALBAC, and MDA were present in Panel A. Among the single-cell libraries, TnBC is closest to bulk genomic DNA, while MDA is the furthest away. Copy number calls on single-cell libraries by TnBC, DOP-PCR, and MALBAC are made accurately (Panel B). TnBC showed the tightest distribution among single-cell methods (Panel C).

Lorenz curves reflect the deviation from perfect uniformity. Because the deviation may come from mutations in genome, as in the case of cancers as well as library preparations, we chose Lorenz curves from normal cells instead of cancerous cells as a basis of comparison to evaluate WGS methods. A subset of Lorenz curves of top performers representing their respective sample/method using 50 kb/bin are presented in Fig 4A. Among all the single-cell libraries, the TnBC is the closest one to the curve of a top performer of bulk libraries, which in turn is the closest one to the perfect uniformity. The differences between TnBC and other methods in Gini index increases as the size of the bins become smaller and smaller (S2 Fig).

#### Evaluation of the uniformity by distribution of copy number (CN) state

Ginkgo [20] is a popular program to predict the CN state of a genome. We used it to analyze the same normal cells from five representative sample/method experiments using 1,000 kb/bin (i.e. an average of 400 reads per bin) as presented in Fig 4B. Except for MDA, the inferred CN states of bulk DNA sample, TnBC, DOP-PCR, and MALBAC matched the genotypes of the cells, 2 for most bins of euchromosomes, and 1 for most bins of the sex chromosomes. The predicted CN state of K562, a leukemia cell line, is quite different from that of the normal cells (S3 Fig), showing multiple alterations of CNs across the genome. The alteration is consistent with the canSAR database (https://cansar.icr.ac.uk/cansar/cell-lines/K-562/copy_number_variation/). The altered CN states of cancer cells argue against that. What we observed from TnBC libraries of normal cells were artifacts.

Although almost all methods gave correct calls on the CN state, the overall confidence level of the call can be quantified by the distribution profile of the inferred CN states (Fig 4C). The bulk DNA has the tightest distribution, while single-cell libraries are more broadly distributed, reflecting that biases were introduced during amplification process. Among them, TnBC and DOP-PCR had their CN state 1 and CN state 2 clearly separated, indicating that an overall high confidence level in copy number calls for both methods. Considering that there is an average of 400 reads per bin, by convention this number confers statistical power to render a confidence level of the CN state call. If the resolution of copy number call is set at 50 kb, then a total of 20 million reads (i.e. about 1X genome coverage) will be needed for the same confidence level.

In our experience, the tightness of the distribution profile of TnBC libraries (Fig 4C) is associated with either early Ct, good distribution of fragment sizes, or optimized PCR protocols.

### Genome evolution during prolonged cell passages

#### Copy number calls

**Fig 5:**
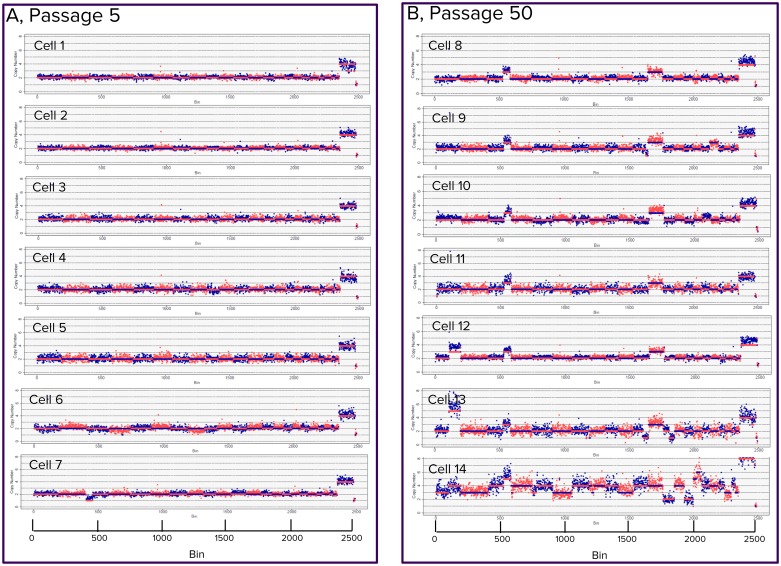
Copy number changes of GM01202 during cell culture. The copy number of GM01202 during a long period of cell culture was monitored using TnBC at Passage 5 (Panel A) and again at Passage 50 (Panel B). At Passage 5, the copy numbers were exactly as reported, while at Passage 50, a region in Chromosome 3 and the whole Chromosome 12 had three copies in six cells of the cells. Two of those six cells also have a common additional extra copy in a region of Chromosome 1. In an extreme example, Cell 14 had a completely different copy number profile.

We applied the TnBC technology in Fluidigm C1 IFCs [29] to investigate copy number changes that were accumulated over a large number of passages. GM01202 is a lymphocyte cell line that has four copies of X, two copies of the euchromsomes, and one copy of Y [30]. We analyzed individual cells at Passage 5 and again at Passage 50 using Mu18 for saturated transposition and sequencing at about 0.1%X coverage. The copy numbers for cells at Passage 5 matched what was reported for these cells [30] and descriptions in the catalog, except that Cell 7 had a deletion in a region of chromosome 3 (Fig 5A). Furthermore, at passage 50, a region in Chromosome 3 and the whole Chromosome 12 had three copies in Cells 8–13. In addition, Cells 12 and 13 had an extra copy in a region of Chromosome 1. Cell 14 exhibited even more complex aneuploidy (Fig 5B). Aneuploidy changes were observed during cell culturing. It has been reported that aneuploidy changes are associated with tumorigenesis and sensitivity to immunotherapy [31–34]. From a practical standpoint, TnBC provides a more economical, more quantitative, more reliable and scalable approach for analyses of cultured cells and cancer cells at finer resolutions in comparison to karyotyping.

#### Confidence level in CNV calls

**Fig 6:**
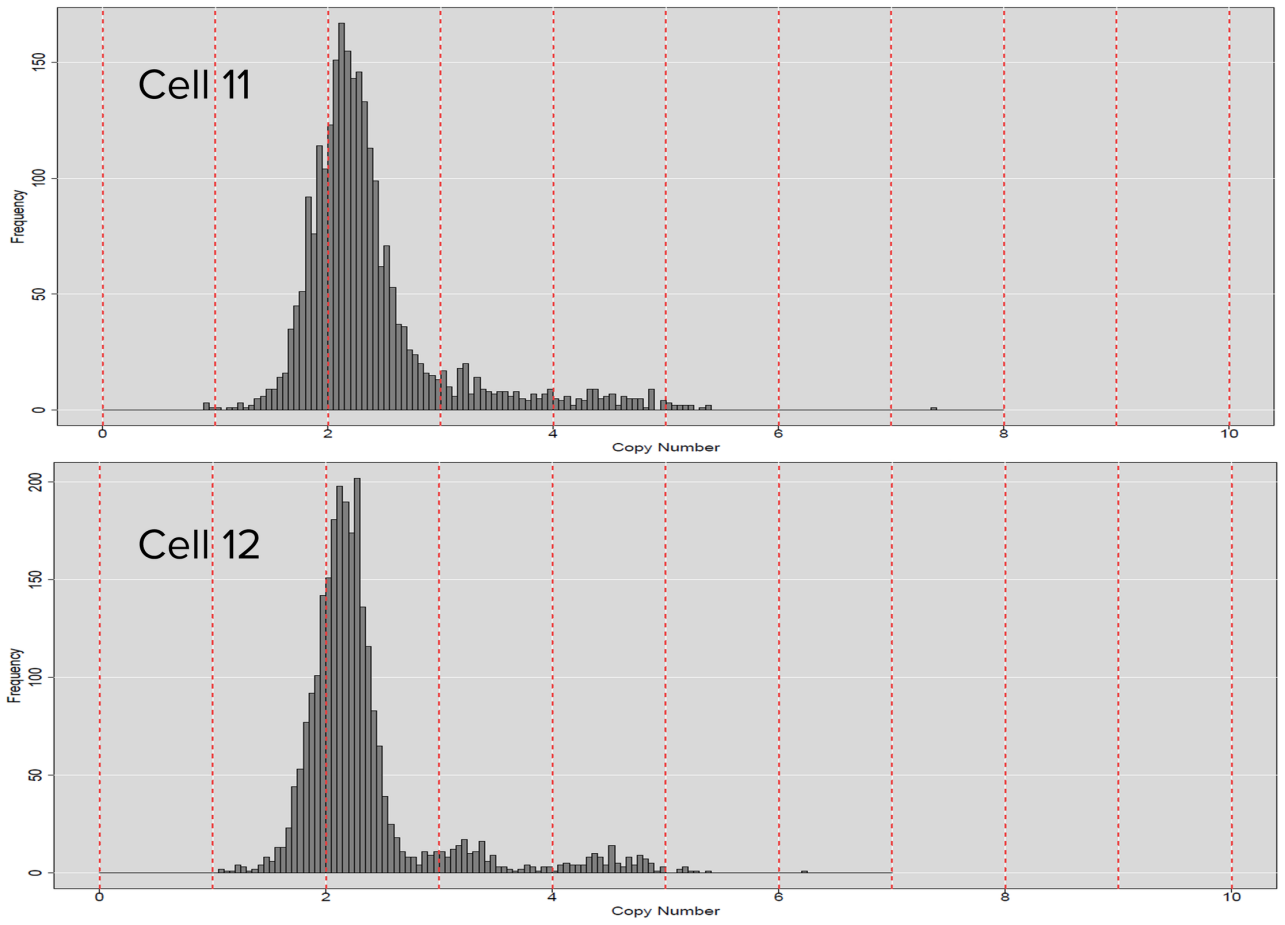
Distribution of CN state of Cell 11 and Cell 12.

Although both Cell 11 and Cell 12 have significant 3-copy calls (Fig 5), clear separation of copies of 2, 3, and 4 is observed for Cell 12, but not for Cell 11 (Fig 6). Based on the principle we laid out in “Evaluation of the uniformity by distribution of copy number (CN) state,” calls on Cell 12 are of higher confidence. The new emerging 3-copy region is clearly between 2-copy and 4-copy peaks in Cell 12 but not in Cell 11.

## Future directions

TnBC library construction involves two steps: saturated transposition and unbiased PCR amplification. The library is quality controlled by Ct, product profile, duplication rate, distribution of CN state, and Lorenz curves. The shallow sequence of the library can be used for revelation of CNV and for a QC step before a commitment to deep sequencing. With optimized protocols, we will apply the methodology to comparison of cancer cells and normal cells. In an ideal TnBC library, each haploid from a single cell will generate one unique set of contiguous fragments. Since each fragment from a set has a unique combination of 5ʹ and 3ʹ ends in the coordinates of the reference genome, two alleles within a cell can be distinguished by their unique 5ʹ and 3ʹ ends combinations, i.e. UFIs (Fig 1). UFIs not only provide barcoding at the individual fragment level, but they also have the potential to be used to link neighboring fragments. When fragments are amplified, the abundance of fragments may vary, but the UFIs remain unchanged (Fig 1). Any variation in abundance of fragments can be normalized using UFIs. With deep sequencing, we expect to detect SNVs and absolute copy number information.

## Supporting information

S1 Fig Lorenz curves of bulk genomic DNA, and of single cells by TnBC, DOP-PCR, MALBAC, and MDA using 10,000K, 5,000K, 2,000K, 1,000K, 500K, 250K, 100K, and 50K variable mapped reads per bin. (TIFF)

S2 Fig Relationship between the Gini index and bin size. Gini indexes of libraries of bulk genomic DNA and of single cells prepared by TnBC, DOP-PCR, MALBAC, and MDA are plotted as a function of the bin size, where the bin contains variable mapped reads. As the size of the bin increase, the Gini index decreased for all libraries.
(TIFF)

S3 Fig Copy number profile of a single cell of K562. (TIFF)
