# Supplementary figures and images for: New library construction method for single-cell genomes
Source: PLoS One. 2017 Jul 19;12(7):e0181163. doi: 10.1371/journal.pone.0181163 (PMC5517011; doi:10.1371/journal.pone.0181163)

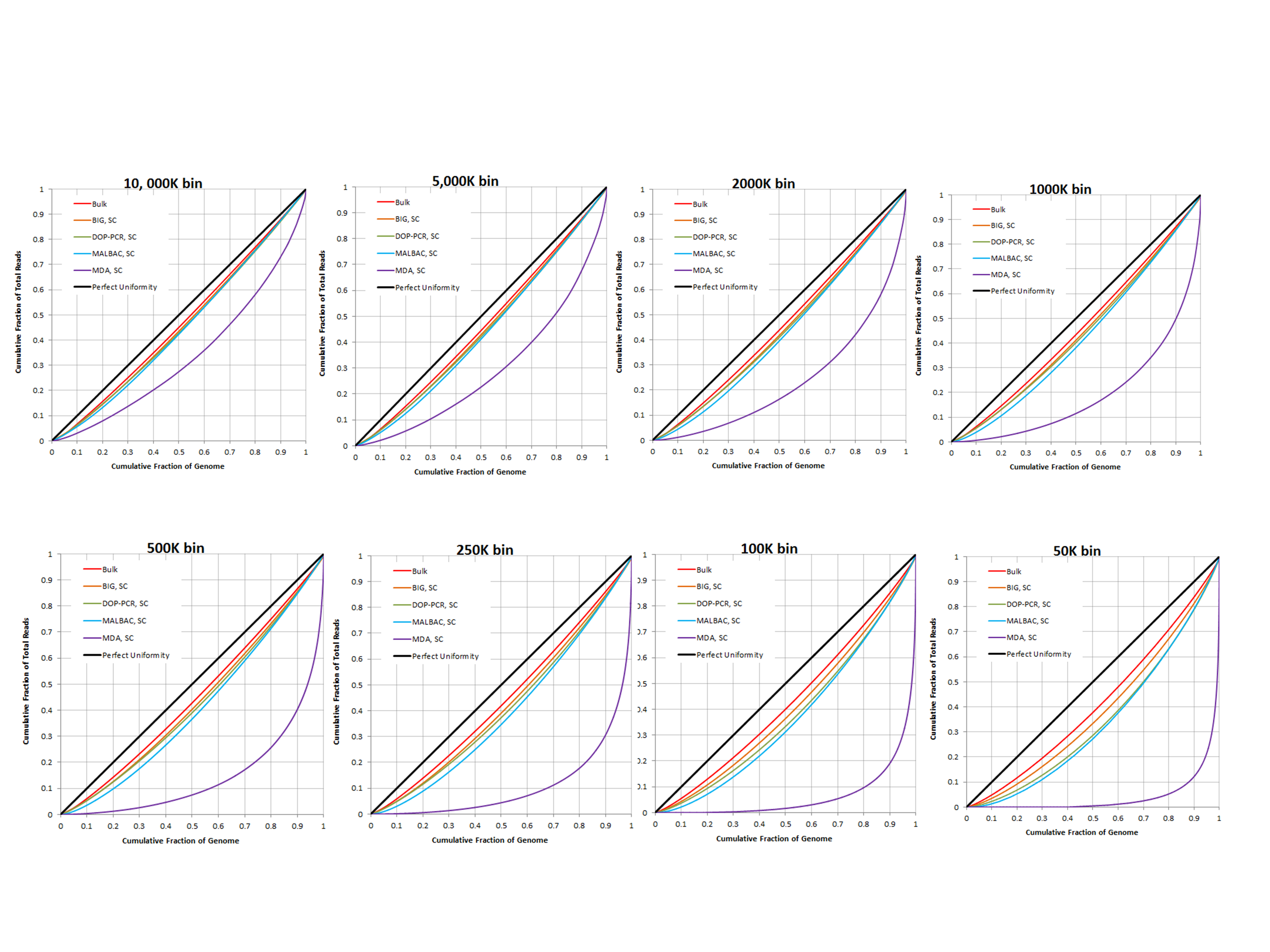

Supplement: S1 Fig — (TIFF) [file pone.0181163.s001.tiff]

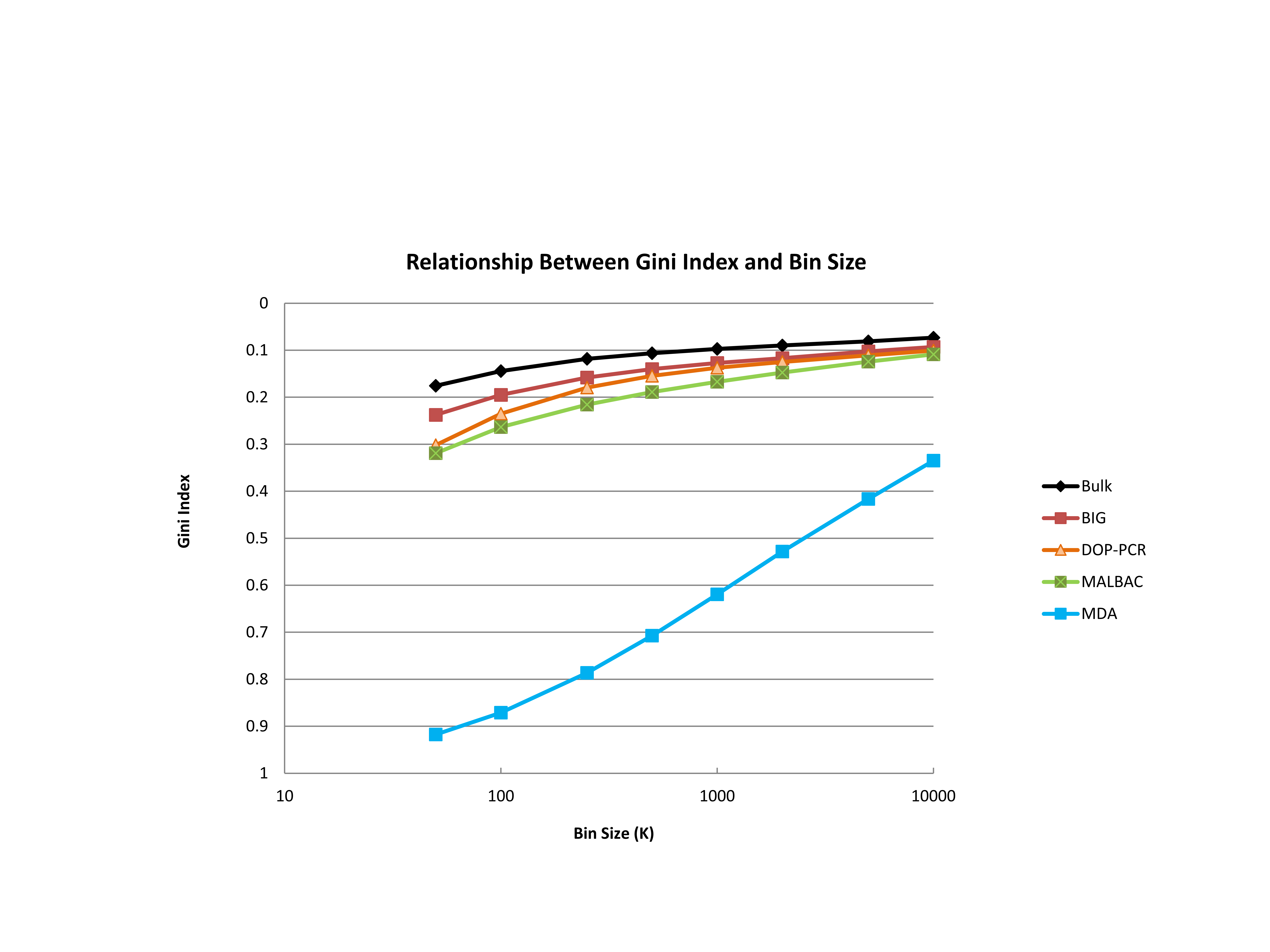

Supplement: S2 Fig — Gini indexes of libraries of bulk genomic DNA and of single cells prepared by TnBC, DOP-PCR, MALBAC, and MDA are plotted as a function of the bin size, where the bin contains variable mapped reads. As the size of the bin increase, the Gini index decreased for all libraries. (TIFF) [file pone.0181163.s002.tiff]

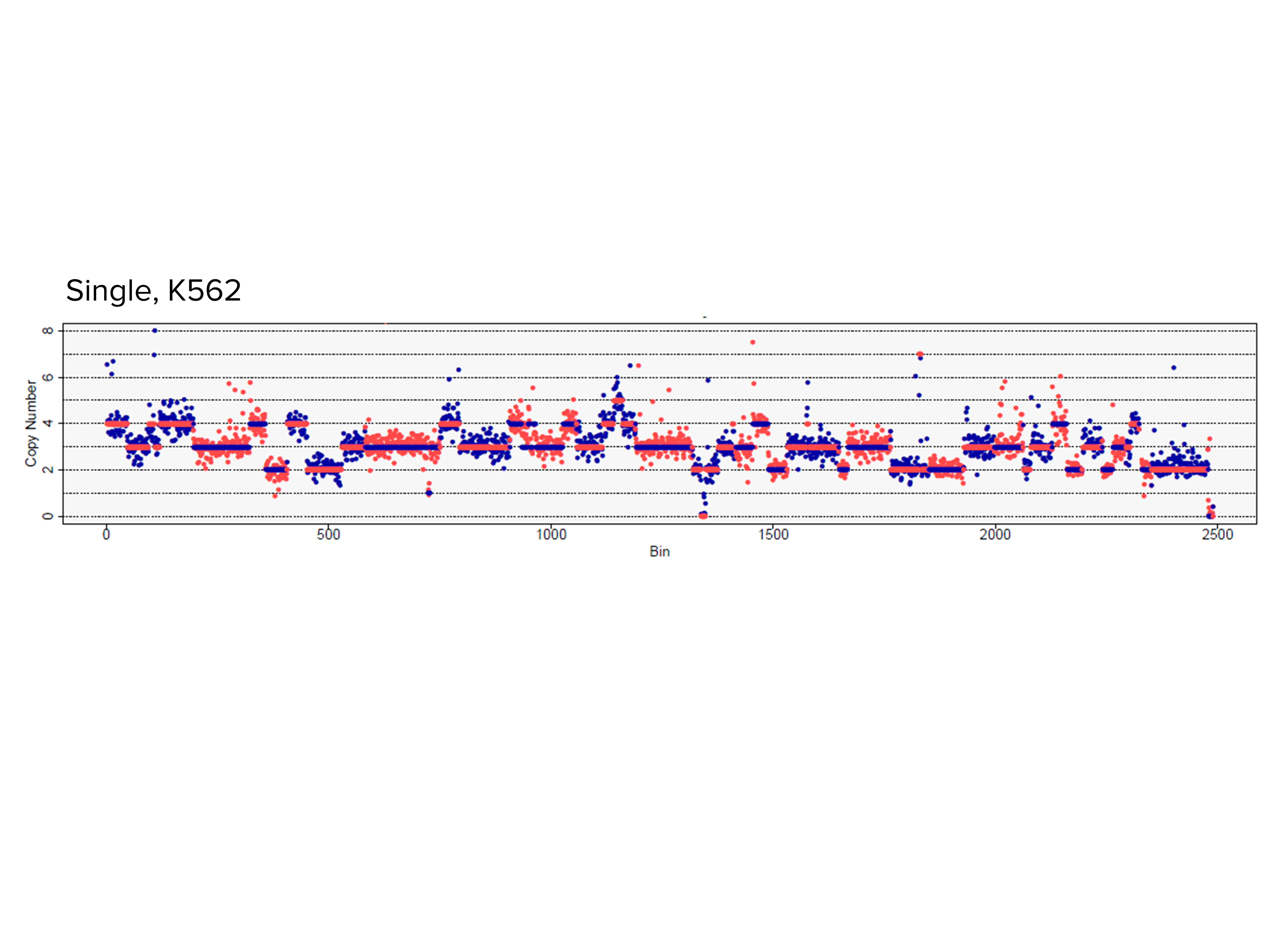

Supplement: S3 Fig — (TIFF) [file pone.0181163.s003.tiff]
